# Dental care trajectories among formerly incarcerated older adults in the United States

**DOI:** 10.1371/journal.pone.0320658

**Published:** 2025-04-08

**Authors:** Alexander Testa, Luis Mijares, Mateus Renno Santos, Vahed Maroufy, Dylan B. Jackson, Rafael Samper-Ternent, Rahma Mungia, Ana C. Neumann

**Affiliations:** 1 Department of Management, Policy and Community Health, School of Public Health, University of Texas Health Science Center at Houston, Houston, Texas, United States of America; 2 Department of Criminology, University of South Florida, Tampa, Florida, United States of America; 3 Department of Biostatistics & Data Science, University of Texas Health Science Center at Houston, Houston, Texas, United States of America; 4 Department of Population, Family, and Reproductive Health, Johns Hopkins Bloomberg School of Public Health, Baltimore, Maryland, United States of America; 5 School of Dentistry, University of Texas Health Science Center at San Antonio, San Antonio, Texas, United States of America; 6 School of Dentistry, University of Texas Health Science Center at Houston, Houston, Texas, United States of America; University of North Carolina at Chapel Hill, UNITED STATES OF AMERICA

## Abstract

A growing body of research has documented an association between prior incarceration and lower dental care use, yet the longitudinal impact of prior incarceration on dental care patterns over time among older adults remains unexplored. This study aims to investigate whether prior incarceration is associated with differing trajectories of dental care use among older adults in the United States. Data were drawn from the 2012-2020 waves of the Health and Retirement Study (HRS), a nationally representative longitudinal survey of older adults in the United States (*n* =  5,893). Group-based trajectory modeling was used to estimate dental care use patterns over time. The findings revealed dental care patterns followed three trajectories: regular dental care use (48.1%), moderate-declining dental care use (27.3%), and low dental care use (24.6%). Multinomial logistic regression was used to examine the relationship between prior incarceration and membership in these trajectory groups. Bivariate analyses revealed that prior incarceration was significantly associated with higher relative risks of being in the moderate-declining (Relative Risk Ratio [RRR] =  2.75, 95% CI =  2.08-3.63) and low dental care use trajectories (RRR =  2.88, 95% CI =  2.10-3.94) compared to the regular dental care use group. After adjusting for sociodemographic, economic, and healthcare-related covariates, formerly incarcerated individuals had a 1.52 times higher relative risk of belonging to the moderate-declining dental care trajectory (RRR =  1.52, 95% CI =  1.16–1.98). The association between prior incarceration and membership in the low dental care trajectory group was not statistically significant. These findings underscore the need for targeted interventions to address dental care disparities among formerly incarcerated individuals, which could lead to improved oral and overall health outcomes for this vulnerable population.

## Introduction

Oral health is critical to overall health, particularly as individuals age into older adulthood [1–3]. Regular dental care can promote oral and overall wellbeing by enabling the prevention, early detection, and treatment of oral diseases [4]. While consistent dental care is important throughout life, it is especially crucial in older adulthood, given the increased risk for tooth decay [5], tooth loss [6], gum disease [7], and oral cancers [8,9]. Even so, around one-third of adults aged 65 and older report not having visited a dentist in the past year, with the rates of not using dental care being particularly elevated among specific populations such as persons of low socioeconomic status and racially minoritized groups [10].

One such population that experiences stark disparities related to dental care use and oral health is formerly incarcerated individuals [4]. Indeed, a growing body of research consistently demonstrates lower rates of dental care utilization among formerly incarcerated adults across the life course, including in early and middle adulthood [11–16] and among older adults [17]. This is particularly concerning considering recent evidence also suggesting that formerly incarcerated older adults are at increased risk of poorer edentulism (i.e., total tooth loss) [18].

Incarceration acts as a significant barrier to dental care through multiple mechanisms. Individuals entering incarceration often come from lower socioeconomic backgrounds [19], where access to and utilization of dental care may already be limited [20–22]. During incarceration, the dental care provided is frequently insufficient and inconsistent, contributing to unmet oral health needs and eroding trust in healthcare systems [20,23,24]. Post-incarceration, these challenges are compounded by additional barriers, such as the stigma associated with a history of incarceration [25,26], persistent mistrust in healthcare [27], financial barriers [28–30], and the lack of insurance [31,32]. Together, these factors perpetuate disparities in dental care access and utilization, further exacerbating oral health inequities among formerly incarcerated individuals.4

Although studies have demonstrated lower dental care use among formerly incarcerated individuals, a significant limitation of extant research is that all prior studies rely on cross-sectional data, where dental care use is observed only at a single time point [11–17]. This approach, while useful, has notable shortcomings because dental care use patterns can vary significantly over the life course [33], and persistently failing to use dental care services year after year may be especially harmful to oral health and overall wellbeing [34]. The current study aims to extend recent cross-sectional research on the relationship between prior incarceration and dental care use among older adults by investigating whether prior incarceration is associated with longitudinal dental care trajectories among older adults over time. We hypothesize that formerly incarcerated individuals will demonstrate patterns of lower dental care use over time compared to those who have never been incarcerated.

## Materials and methods

The data for this study are from the Health and Retirement Study (HRS), a large, ongoing, nationally representative biennial longitudinal survey of Americans aged 50 and older, that is sponsored by the National Institute on Aging (grant number NIA U01AG009740) and is conducted by the University of Michigan [35]. In 2012 and 2014, the survey included questions regarding respondents’ incarceration history, which were collected through a leave-behind questionnaire (LBQ) that participants returned by mail. Half of the study participants were selected to respond to the LBQ in 2012, and the other half in 2014 [36–38]. The 2012 LBQ data is representative of those aged 53 and older, while in 2014, the LBQ data represents those 55 and older. Accordingly, the current study uses adults aged 55 and older who completed the LBQ and participated in the 2012-2020 HRS (*n* =  5,893). Prior to participation in each HRS interview, participants are provided with a written informed consent information document.

At the start of each interview, all respondents read a confidentiality statement and gave oral consent by agreeing to do the interview [39]. The University of Texas Health Science Center at Houston Committee for the Protection of Human Subjects deemed this study exempt from human subjects review because the HRS data are publicly accessible and de-identified (Reference #263855). Further details and access to the data can be found on the HRS website maintained by the University of Michigan: https://hrs.isr.umich.edu. Results are reported using the Strengthening the Reporting of Observational Studies in Epidemiology (STROBE) reporting guidelines for cohort studies.

### Dependent variable

The primary outcome variable, *dental care use*, was assessed by asking respondents, “[i]n the last two years have you seen a dentist for dental care, including dentures?” (yes or no) [17,40]. To estimate trajectories of dental care use, we used responses from 2012 (the first year of LBQ) to 2020 (the most recent year of data available at the time of analysis).

### Exposure variable

The exposure variable, *prior incarceration*, was measured with a question asking, “[h]ave you ever been an inmate in jail, prison, juvenile detention center, or other correctional facility.” (yes or no) [17,36,37,41].

### Covariates

Several variables previously associated with incarceration and health-related outcomes in research using the HRS data were included as covariates. All covariates are measured when the respondent answered the LBQ (2012 or 2014). Demographic control variables included *race/ethnicity* (Hispanic, non-Hispanic Black, non-Hispanic other, or non-Hispanic White) and *biological sex* (male or female) due to racial/ethnic and sex disparities in incarceration and dental care use [42,43]. Covariates are also included to account for *educational attainment* (less than high school, GED, high school graduate, some college, or college degree and above), *veteran status* (yes or no), and whether the respondent’s *mother graduated high school* (yes or no). Additionally, the analysis accounted for respondents’ general use of healthcare services by including variables indicating whether they had *visited a medical doctor* in the past two years (yes or no) and whether they had stayed *overnight in a hospital* during the same period (yes or no). A control for *wealth* was determined by calculating the net value of all non-housing assets (e.g., stocks, mutual funds, trusts, checking and savings accounts, bonds, and other financial resources) minus total debts. Following prior studies utilizing the HRS data, wealth was divided into four quartiles, from the lowest (1st quartile) to the highest (4th quartile) [36]. Finally, *dental insurance coverage* was assessed by asking respondents, “do you have any insurance that covers your dental bills?” (yes or no).

## Statistical analysis

Longitudinal patterns of dental care use from 2012-2020 were estimated using group-based trajectory modeling (GBTM), a semi-parametric longitudinal latent class modeling technique to examine distinct patterns of behavior across time [44,45]. GTBM uses maximum likelihood estimation to assign individuals to the trajectory group that best represents their observed data based on the greatest statistical probability of group membership. Given the binary nature of the dental care use variable, trajectories are estimated using a logistic functional form using the “traj” package in STATA (Version 18) [46]. In all models, we use an extension described by Haviland et al [47]. to account for nonrandom attrition. The probability of attrition, which rises alongside respondent age, is presented in Appendix A in S1 File.

We estimated trajectories starting with a two-group model, incrementally adding additional groups until adding another group, and evaluating model fit using diagnostic criteria such as the Bayesian Information Criterion (BIC), transformed Bayes factor, and Entropy. While these diagnostic criteria are an important consideration, the ultimate selection of a model was made by balancing multiple factors, including identifying meaningful trajectories, ensuring model simplicity, adequate statistical power for each trajectory group, and achieving model adequacy. Once the number of groups was established, we refined the model by adjusting the functional forms of the groups (e.g., zero-order, linear, quadratic, polynomial cubic) to identify the best-fitting model. A final three-group solution was selected by balancing the statistical diagnostic criteria (i.e., BIC), while ensuring each trajectory group had a sufficient sample size [44].

After estimating the trajectory model, we classified respondents to the trajectory of which they had the highest statistical probability of membership and used multinomial logistic regression to assess the relationship between prior incarceration measured at the 2012 or 2014 LBQ and dental care use trajectory group membership. Analyses were adjusted for survey weights (*llbwgtr)*, clustering (*raehsamp*), and stratum (*raestrat*) information using the *syv* command in Stata (Version 18) [48].

## Results

Table 1 provides the summary statistics for the analytic sample, demonstrating that 6.5% of respondents (n =  229) reported prior incarceration. The sample composition was 46.3% (n =  2,477) male, 7.8% Hispanic (n =  612), 9.0% non-Hispanic Black (n =  873), 3.1% non-Hispanic other race (n =  162), and 80.1% non-Hispanic White (n =  4,226).

**Table 1:** Summary statistics from the health and retirement study (unweighted N =  5,893; weighted N =  34,578,965).

Variables		Weighted %/Mean[SD]	Unweighted Frequency
Previous Incarceration	No	93.5	5,529
	Yes	6.5	229
Age Categories	55-64	50.8	2,391
	65-74	31.3	1,982
	75-84	15.9	1,391
	85^ + ^	2.03	129
Sex	Female	53.7	3,416
	Male	46.3	2,477
Race and Ethnicity^a^	Hispanic	7.8	612
	Non-Hispanic Black	9.0	873
	Non-Hispanic Other^b^	3.1	162
	Non-Hispanic White	80.1	4,226
Educational Attainment	Less Than High School	11.2	820
	GED/High School	31.9	2,028
	Some College	26.4	1,504
	College Graduate	30.5	1,541
Veteran Status	No	80.9	4,749
	Yes	19.1	1,144
Mother’s Education	Less Than High School	42.4	2,889
	High School Graduate	57.6	2,995
Hospital Stay	No	73.0	4,172
	Yes	27.0	1,721
Visited Doctor	No	7.3	479
	Yes	92.7	5,414
Quantile of Wealth	1st Quantile	14.0	970
	2nd Quantile	21.7	1,368
	3rd Quantile	27.5	1,626
	4th Quantile	36.7	1,929
Dental Insurance	No	52.0	3,356
	Yes	48.0	2,537

^a^Race and ethnicity were self-reported by survey respondents.

^b^Non-Hispanic Other category included American Indian or Alaskan Native, Asian, or Pacific Islander.

**Fig 1:**
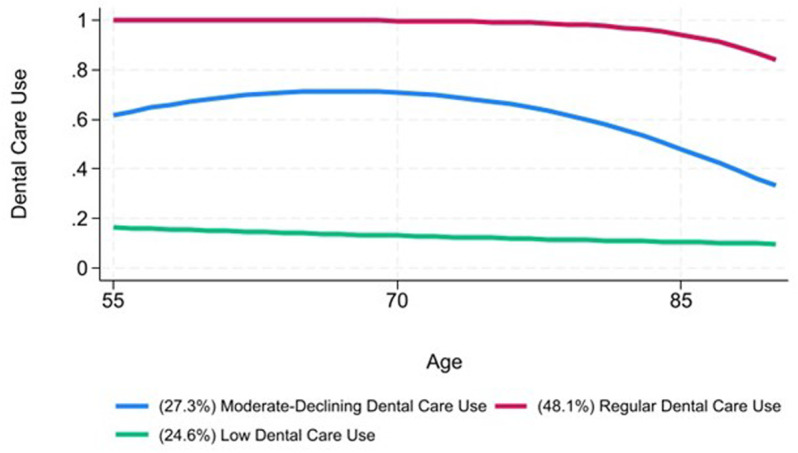
Results of the Group Based Trajectory Model of Dental Care Use in the 2012-2020 HRS Survey.

Fig 1 presents the results of the GBTM analysis of dental care use trajectories spanning ages 55 to 90 from the 2012-2020 HRS surveys. The best-fitting model identified three distinct trajectory groups, consisting of one quadratic trajectory and two linear trajectories. Model fit statistics, presented in Appendix B in S1 File, indicate strong model fit. The average posterior probability (APP) for all groups exceeded the recommended threshold of 0.70, and the odds of correct classification (OCC) surpassed the suggested minimum value of 5. [44]. Additionally, the 3-group model reported an Entropy of.655. Notably, while there is consensus that higher Entropy values indicate better class separation, there are no universally agreed-upon cutoffs for their interpretation [49,50]. For the current study, the Entropy exceeds the threshold of 0.5 as recommended by some scholars [51]. but remains below the stricture thresholds of > 0.80 as recommended by others [52].

Though these groups are latent approximations, we developed descriptive labels to aid with the interpretation of the results [44]. The largest group, *Regular Dental Care*, includes 48.1% (*n* =  2,842) of the sample and is characterized by individuals who consistently use dental care services but experience a slight decline around the time they reach their mid-80s. Next, *Moderate-Declining Dental Care Use* includes 27.3% (*n* =  1,498) of the sample and is characterized by moderate dental care use patterns that remain relatively stable from ages 55 until the mid-70s, when care begins to decline steeply. Finally, the *Low Dental Care* group encompasses 24.6% (*n* =  1,553) of the sample and is characterized by respondents with consistently low levels of dental care use, which steadily decline with age.

Table 2 presents the multinomial logistic regression analysis estimating the association between prior incarceration and dental care use trajectories, with *Regular Dental Care* set as the base category. First, in Model 1, we assess the bivariate relationship between prior incarceration and dental care use trajectories, finding that formerly incarcerated individuals have a 2.75 times higher relative risk of membership. in the *Moderate-Declining Dental Care Use* (Relative Risk Ratio [RRR] =  2.75, 95% Confidence Interval [CI] =  208-3.63) compared to the *Regular Dental Use* trajectory and a 2.88 times higher relative risk of membership in the *Low Dental Care Use* (RRR =  2.88, 95% CI =  2.10-3.94) compared to the *Regular Dental Care Use* trajectory. Next, in Model 2, we adjust for a series of covariates and find that prior incarceration is associated with a 1.82 times higher relative risk of membership in *Moderate-Declining Dental Care Use* trajectory compared to the *Regular Dental Care Use* trajectory (RRR =  1.82, 95% CI =  1.37-2.41) and a 1.55 times higher relative risk of membership in the *Low Dental Care Use* compared to the *Regular Dental Care Use* trajectory (RRR =  1.55, 95% CI =  1.06-2.28). Finally, we adjust for baseline wealth and dental insurance status, finding prior incarceration is associated with a 1.52 times higher relative risk of membership in the *Moderate-Declining Dental Care Use* trajectory compared to *Regular Dental Care Use* trajectory (RRR =  1.52, 95% CI =  1.16-1.98). However, there is no longer a significant association between prior incarceration and membership in the *Low Dental Care* trajectory.

**Table 2:** Multinomial logistic regression of dental care use trajectories on prior incarceration and covariates (unweighted N =  5,893; weighted N =  34,578,965).

Variables	Model 1		Model 2		Model 3	
	Moderate-Declining Dental Care Use vs Regular Dental Care	Low Dental Care Use vs Regular Dental Care	Moderate-Declining Dental Care Use vs Regular Dental Care	Low Dental Care Use vs Regular Dental Care	Moderate-Declining Dental Care Use vs Regular Dental Care	Low Dental Care Use vs Regular Dental Care
	OR (95% CI)	OR (95% CI)	RRR (95% CI)	RRR (95% CI)	RRR (95% CI)	RRR (95% CI)
Prior Incarceration	**2.75 (2.08-3.63)**	**2.88 (2.1-3.94)**	**1.82 (1.37-2.41)**	**1.55 (1.06-2.28)**	**1.52 (1.16-1.98)**	1.10 (0.75-1.59)
Age - 55-64 (Reference)	NA	NA	NA	NA	NA	NA
Age - 65-74	NA	NA	1.04 (0.87-1.26)	0.92 (0.74-1.14)	0.99 (0.83-1.18)	0.88 (0.71-1.10)
Age - 75-84	NA	NA	**0.75 (0.58-0.96)**	0.89 (0.72-1.11)	**0.65 (0.51-0.83)**	**0.74 (0.78-0.97)**
Age - 85^ + ^	NA	NA	**0.13 (0.06-0.28)**	0.88 (0.71-1.09)	**0.10 (0.05-0.22)**	**0.31 (0.17-0.58)**
Male	NA	NA	**1.39 (1.13-1.72)**	**0.44 (0.26-0.74)**	**1.56 (1.24-1.95)**	**2.21 (1.74-2.80)**
Non-Hispanic White (Reference)	NA	NA	NA	NA	NA	NA
Non-Hispanic Black	NA	NA	**3.27 (2.49-4.30)**	**2.69 (1.97-3.67)**	**2.52 (1.87-3.39)**	**1.66 (1.20-2.28)**
Hispanic	NA	NA	**2.10 (1.41-3.11)**	1.51 (0.93-2.45)	**1.89 (1.26-2.84)**	1.21 (0.76-1.92)
Non-Hispanic Other	NA	NA	1.76 (0.97-3.20)	1.44 (0.83-2.50)	1.51 (0.79-2.88)	1.03 (0.58-1.82)
Less than high school (Reference)	NA	NA	NA	NA	NA	NA
GED/High School	NA	NA	**0.74 (0.55-1.00)**	**0.41 (0.30-0.55)**	0.90 (0.67-1.23)	**0.56 (0.41-0.77)**
Some College	NA	NA	**0.56 (0.39-0.79)**	0.24 (0.17-0.33)	0.77 (0.54-1.10)	**0.40 (0.29-0.55)**
College Graduate	NA	NA	**0.27 (0.19-0.38)**	**0.06 (0.05-0.08)**	**0.45 (0.31-0.64)**	**0.15 (0.11-0.20)**
Military Veteran	NA	NA	0.80 (0.61- 1.05)	1.04 (0.81-1.33)	0.78 (0.60-1.01)	0.97 (0.73-1.29)
Mother High School Graduate	NA	NA	0.85 (0.68 - 1.05)	**0.75 (0.62-0.90)**	0.95 (0.77-1.17)	0.88 (0.71-1.10)
Hospital Stay - Past 2 Years	NA	NA	**1.21 (1.00-1.48)**	**1.51 (1.29-1.76)**	1.06 (0.86-1.31)	1.18 (0.98-1.42)
Visited Doctor - Past 2 Years	NA	NA	**0.40 (0.29-0.55)**	**0.34 (0.24-0.48)**	**0.40 (0.28-0.58)**	**0.34 (0.23-0.51)**
Wealth - 1st Quantile (Reference)	NA	NA	NA	NA	NA	NA
Wealth -2nd Quantile	NA	NA	NA	NA	0.89 (0.64-1.25)	**0.57 (0.42-0.78)**
Wealth -3rd Quantile	NA	NA	NA	NA	**0.45 (0.32-0.64)**	**0.22 (0.15-0.32)**
Wealth - 4th Quantile	NA	NA	NA	NA	**0.30 (0.21-0.43)**	**0.08 (0.06-0.11)**
Dental Insurance	NA	NA	NA	NA	**0.49 (0.28-0.58)**	**0.27 (0.22-0.33)**

*Note***:** Boldface denotes statistical significance at *p* < .05 level.

### Supplementary analyses

Supplemental analyses were conducted using a question on *incarceration duration*, asking respondents, “[i]n your entire life, how much time in total have you been detained in a jail, prison, juvenile detention center, or other correctional facility” (Less than one month, less than one year, between 1-5 years, more than 5 years). To ensure adequate cell sizes for analyses and consistent with categorization in prior research [36], the response options were classified as: (a) never incarcerated, (b) less than one month, (c) more than one month. The results in Appendix B in S1 File revealed that net of covariates, those who have been previously incarcerated for more than 1 month had approximately a 2.6 times greater relative risk of membership in the *Moderate-Declining Dental Care Use* trajectory compared to the *Regular Dental Care Use* trajectory, relative to never incarcerated respondents (RRR =  2.63, 95% CI =  1.31-5.32). We found no significant differences in dental care between respondents who were incarcerated for less 1 month and those who were never incarcerated.

## Discussion

The results of this study demonstrate a relationship between prior incarceration and dental care use trajectories among older adults, revealing that formerly incarcerated individuals are more likely to follow patterns of moderate-declining dental use trajectories compared to regular dental care use. These findings are consistent with previous cross-sectional studies that have established a link between prior incarceration and lower dental care utilization in early and middle adulthood [11–16], as well as recent research using the HRS to examine dental care use among older adults at a single point in time [17]. The current study extends this prior literature by conducting the first longitudinal investigation of dental care use among older formerly incarcerated adults to the authors’ knowledge, thereby demonstrating that the association between prior incarceration and less frequent dental care use is sustained over an extended period.

Our research extends existing research on incarceration’s adverse health effects to include long-term oral health disparities. Specifically, this is only the second study to investigate the association between incarceration and dental care for older adults [17], while it is the first study to explore this association from a longitudinal perspective. Oral health is sensitive to regular preventive care, and the consequences of negligence may be both severe and permanent, including aesthetic loss, loss of function, pain, and related health conditions—all of which can be profoundly distressing [53,54]. Neglecting dental care not only results in severe oral damage but can also lead to the development of further health issues, including systemic conditions such as cardiovascular diseases and diabetes [55]. Considering the extraordinarily high levels of incarceration in the US, and the fact these levels have been high for nearly 50 years [42], an enormous contingent is subject to these consequences linked to their experience with incarceration. Specifically, best estimates indicate that approximately 1-in-15 adults over age 50 have a history of incarceration [36]. Our trajectory model indicates that these individuals will be much less likely to meet the recommendations of biannual dental care visits [56], and that their dental care use is likely to worsen as they age, particularly after age 75. Furthermore, the study findings demonstrate that formerly incarcerated individuals exhibit lower dental care use, even adjusting for wealth level and dental insurance, indicating that barriers to care extend beyond financial constraints and may encompass factors such as stigma, lack of access, and mistrust [16,25,27,57]. This pattern of low dental care use can impact the quality of life for formerly incarcerated older adults, as issues such as periodontal disease, tooth decay, and chronic pain can affect daily activities and place an additional burden on these individuals, further exacerbating their overall health challenges [58].

These findings point to important implications for oral health and overall wellbeing among formerly incarcerated individuals and highlight potentially relevant areas for public health practice and intervention. Prior to incarceration, interventions targeting populations at high risk for incarceration could promote preventive care and reduce the severity of oral health issues. The period of incarceration could serve as an opportunity to improve dental care by offering consultations and programs to address existing oral health issues and promote dental hygiene, including brushing, flossing, and regular dental visits. Preventative dental care programs for incarcerated individuals can reduce the prevalence of common oral health issues like periodontal disease and tooth decay, which result from limited access to care. Early intervention before release can potentially lower long-term dental costs by preventing more complex and expensive procedures, ultimately alleviating the burden on public health systems and individuals [59]. Though these interventions should be subject to empirical tests, it is plausible that by improving oral health care and access before or during incarceration, formerly incarcerated individuals may continue to experience improved oral health and dental care use following the release of oral health by offering expanded care and information to patients, especially when these programs are coupled with access and care coordination to dental care services post-release [60].

Finally, interventions can also focus on bolstering access to dental care post-release. To the extent that issues with dental care access are the underlying mechanism in the relationship between prior incarceration and dental care use, the findings point to the need to integrate dental care within broader public health initiatives, discharge planning from detention, and community reintegration programs for formerly incarcerated individuals could be a key area for research to tackle the interconnected issues of dental care access and social reintegration [61]. One potential means of doing so is to expand existing programs such as Transition Clinic Network—a national network of primary care clinics that provides patient-centered and culturally appropriate care for people with a history of incarceration—to be leveraged and expanded to enhance dental care use as a means of increasing dental care uptake for formerly incarcerated individuals over the lifespan [62].

## Limitations

This study has limitations that should be considered when interpreting the findings. First, prior incarceration is measured via a self-report and may be subject to measurement error due to recall or social desirability bias. Second, the measure of incarceration also lacked potentially important details related to a respondent’s incarceration experience, such as how many times an individual was incarcerated, the type of facility, when in the life course incarceration(s) took place, time since release, and specific experiences during incarceration, including with dental or healthcare services. Third, the measure of dental care use in the HRS data does not include the reason for a dental visit, including whether it is for preventive or restorative dental care. Fourth, the measurement of dental care was a binary variable, which may limit the ability to capture nuanced variations within the data, as it reduces complex behaviors or outcomes into a simple dichotomy. Future research that includes more detailed measures of the frequency of dental care use each year and the count of different procedures received can provide a more detailed picture of how older formerly incarcerated adults use (or do not use) dental care over time. Fifth, there may be unobserved variables that potentially explain the relationship between prior incarceration and dental care use, such as variation in neighborhood socioeconomic conditions or geographic barriers to accessing dental care, which could further explain disparities in dental care. Future research should aim to collect and analyze multi-level longitudinal data to understand how incarceration influences dental care use across the life course.

## Conclusions

Using nationally representative data, the current study provides the first longitudinal evidence about the relationship between prior incarceration and patterns of dental care use. Findings demonstrate that formerly incarcerated older adults are significantly more likely to have dental care use trajectories over an 8-year period that are marked by less regular dental care. The study underscores the importance of research exploring the underlying mechanisms that contribute to the lower utilization of dental care services among formerly incarcerated older adults. Further understanding these factors is essential for developing informed programmatic and policy efforts to effectively increase dental care use within this population, ultimately advancing health equity and improving oral and overall health outcomes.

## Supporting information

S1 File Appendices A to C. (DOCX)

S1 Fig Appendix figure. (JPG)
