# Supplementary material for: Dental care trajectories among formerly incarcerated older adults in the United States
Source: PLoS One. 2025 Apr 8;20(4):e0320658. doi: 10.1371/journal.pone.0320658 (PMC11978076; doi:10.1371/journal.pone.0320658)
Supplement: S1 File — (DOCX) [file pone.0320658.s001.docx]

**Appendix A: Probability of Attrition in the 2012-2020- HRS Survey**

**Appendix B: Model Fit Statistics**

| **Model** | **BIC** | **2loge(B10)^2^** | **Group Membership** | **APP** | **OCC** | **Entropy** |
| --- | --- | --- | --- | --- | --- | --- |
| Model 1, 1 Group | -26636.01 |  | G1: 100.00% | 1 | 1 |  |
| Model 2, Groups | -20105.61 | 13060.8 | G1: 38.60% G2: 61.40% | G1: 0.92% G2: 0.94% | G1: 35.79 G2: 36.52 | .763 |
| Model 3, 3 Groups | -19866.87 | 477.48 | G1: 25.98% G2: 50.96% G3: 23.06% | G1: 0.73% G2: 0.89% G3: 0.80% | G1: 16.63 G2: 23.3 G3: 20.64 | .612 |
| Model 4, 4 Groups | -19876.95 | -20.16 | G1: 10.51% G2: 17.72% G3: 49.12% G4: 22.65% | G1: 0.47% G2: 0.53% G3: 0.86% G4: 0.76% | G1: 59.36 G2: 9.01 G3: 17.87 G4: 14.63 | .544 |
| Model 5, 5 Groups | -19844.76 | 64.38 | G1: 22.66% G2: 25.44% G3: 8.31% G4: 36.0% G5: 7.59% | G1: 0.63% G2: 0.75% G3: 0.61% G4: 0.68% G5: 0.53% | G1: 7.45 G2: 25.65 G3: 354.96 G4: 6.77 G5: 47.8 | .524 |
| Model 6, 6 Groups | -19804.5 | 80.52 | G1: 2.93% G2: 13.19% G3: 17.53% G4: 37.24% G5: 18.18% G6: 10.92% | G1: 0.52% G2: 0.52% G3: 0.58% G4: 0.66% G5: 0.63% G6: 0.50% | G1: 306.13 G2: 38.97 G3: 16.15 G4: 5.63 G5: 8.44 G6: 39.05 | .480 |
| Model 7, 7 Groups | -19791.19 | 26.62 | G1: 2.95% G2: 16.93% G3: 10.79% G4: 21.87% G5: 22.86% G6: 12.13% G7: 12.47% | G1: 0.53% G2: 0.57% G3: 0.53% G4: 0.49% G5: 0.45% G6: 0.46% G7: 0.54% | G1: 359.45 G2: 14.29 G3: 44.11 G4: 9.2 G5: 3.67 G6: 12.28 G7: 10.22 | .427 |
| Selected Model:  order (2,1,1) | -10903 |  | G1: 29.52% G2: 46.6% G3: 23.88% | G1: 0.83% G2: 0.87% G3: 0.86% | G1: 14.81 G2: 7.41 G3: 16.73 | .655 |

*Note*: 2loge(B10)^2 is^ not included in the selected model because it is the metric of the change in the BIC between orders.

**Appendix C: Multinomial Logistic Regression of Dental Care Use Trajectories on Prior Incarceration Length and Covariates (Unweighted N = 5,876; Weighted N = 34,496,965)**

| **Variables** | **Moderate Declining Dental Care Use vs**  **Regular Dental Care Use** | **Low Dental Care Use vs**  **Regular Dental Care Use** |
| --- | --- | --- |
|  | **RRR (95% CI)** | **RRR (95% CI)** |
| Never incarcerated (Reference) | NA | NA |
| Less than 1 month incarcerated | 1.30 (0.89 - 1.89) | 1.00 (0.59-1.66) |
| More than 1 month incarcerated | **2.63 (1.31 - 5.32)** | 1.02 (0.76 - 3.47) |

Boldface denotes statistical significance at *p*<.05 level.

*Note:* Control variables include age, race/ethnicity, educational attainment, veteran status, mother's education, hospital stay – past 2 years, visited doctor – past 2 years, wealth quartile, and dental insurance
