# Supplementary figures and images for: Dental care trajectories among formerly incarcerated older adults in the United States
Source: PLoS One. 2025 Apr 8;20(4):e0320658. doi: 10.1371/journal.pone.0320658 (PMC11978076; doi:10.1371/journal.pone.0320658)

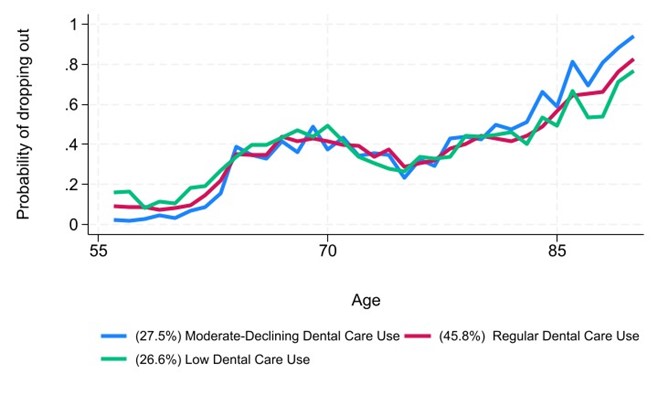

Supplement: S1 Fig — (JPG) [file pone.0320658.s002.jpg]
